# Multiple episodes of aspirin overdose in an individual patient: a case report

**DOI:** 10.1186/1752-1947-8-374

**Published:** 2014-11-19

**Authors:** Debasish Ghosh, Kenneth M Williams, Garry G Graham, Priya Nair, Hergen Buscher, Richard O Day

**Affiliations:** 1 Department of Clinical Pharmacology and Toxicology, St Vincent’s Hospital, 390 Victoria Street, Darlinghurst, Sydney, NSW 2010, Australia; 2 Intensive Care Unit, St Vincent’s Hospital, 390 Victoria Street, Darlinghurst, Sydney, NSW 2010, Australia; 3 St Vincent’s Clinical School, University of New South Wales, Sydney, NSW, Australia

**Keywords:** Aspirin, Euvolemia, Overdose, Potassium, Salicylate, Toxicity, Urinary alkalization

## Abstract

**Introduction:**

Aspirin overdose, though now infrequently encountered, nevertheless continues to contribute to significant morbidity and mortality. The patient described in this case report intentionally ingested overdoses of aspirin on repeated occasions. The case provided an unusual and possibly one-of-a-kind opportunity to focus on the variability in the time course of plasma salicylate concentrations with current treatment modalities of aspirin overdose in an individual patient.

**Case presentation:**

A 75-year-old Caucasian man who weighed 45kg and had an extensive history of various drug overdoses and stage 3 chronic kidney disease presented to a tertiary university hospital on three occasions within 2 months after successive overdoses of aspirin. During his third admission, he overdosed with aspirin, while on the ward recovering from the previous aspirin overdose. The overdoses were categorized as “potentially lethal” on two occasions and as “serious” in the other two, based on the alleged dose of aspirin ingested (over 500mg/kg in the first two overdoses, and 320mg/kg and 498mg/kg in the other two, respectively). However, as assessed by the observed salicylate concentrations, the ingestions would more appropriately have been categorized as being of “moderate” severity for the first and second overdose and “mild” severity for each of the others. This categorization was more consistent with the clinical severity of his admissions. A single dose of activated charcoal was administered only after the second overdose. On each occasion, he was given intravenous fluid with the aim of achieving euvolemia. Urinary alkalization was not attempted during the first admission, which was associated with the longest apparent elimination half-life of salicylate (30 hours). A plasma potassium concentration of approximately 4mmol/L appeared to be needed for adequate urinary alkalization.

**Conclusion:**

In a patient with impaired renal function, intravenous fluid and urinary alkalization are the mainstays of treatment of aspirin overdose. Correction of hypokalemia is recommended. Repeated doses of charcoal may be a worthwhile intervention when there is no risk of aspiration. Our experience in this case also revealed considerable unexplained variation in management despite the availability of guidelines. It is, therefore, important to monitor the implementation of available guidelines.

## Introduction

Aspirin is readily available as an over-the-counter (OTC) medication. Use of this drug has decreased because of the availability of newer anti-inflammatory drugs that are better tolerated for the treatment of musculoskeletal pain and inflammation. Evidence of an association between aspirin and Reye’s syndrome has also contributed significantly to decreasing aspirin use in children. Strategies such as child-resistant packaging and reducing the amount of medication in each package of OTC analgesics have also contributed to a decrease in the incidence of aspirin poisoning [1]. Despite its decreasing incidence, aspirin poisoning remains an important clinical problem involving accidental ingestion in children and intentional overdose in adults [2]. Salicylate, a metabolite of aspirin, seems to be responsible for the toxic effects of the drug [3].

We present the case of a patient who presented with aspirin overdoses on four occasions over the course of two months at a tertiary university hospital. Our aim in this report was to use the unusual and possibly one-of-a-kind opportunity to describe repeated overdose in the same individual with a focus on (1) the present treatment modalities for aspirin overdose and (2) the variability in the time course of plasma salicylate concentrations between episodes and the potential influences of therapeutic interventions.

## Case presentation

**Figure 1:**
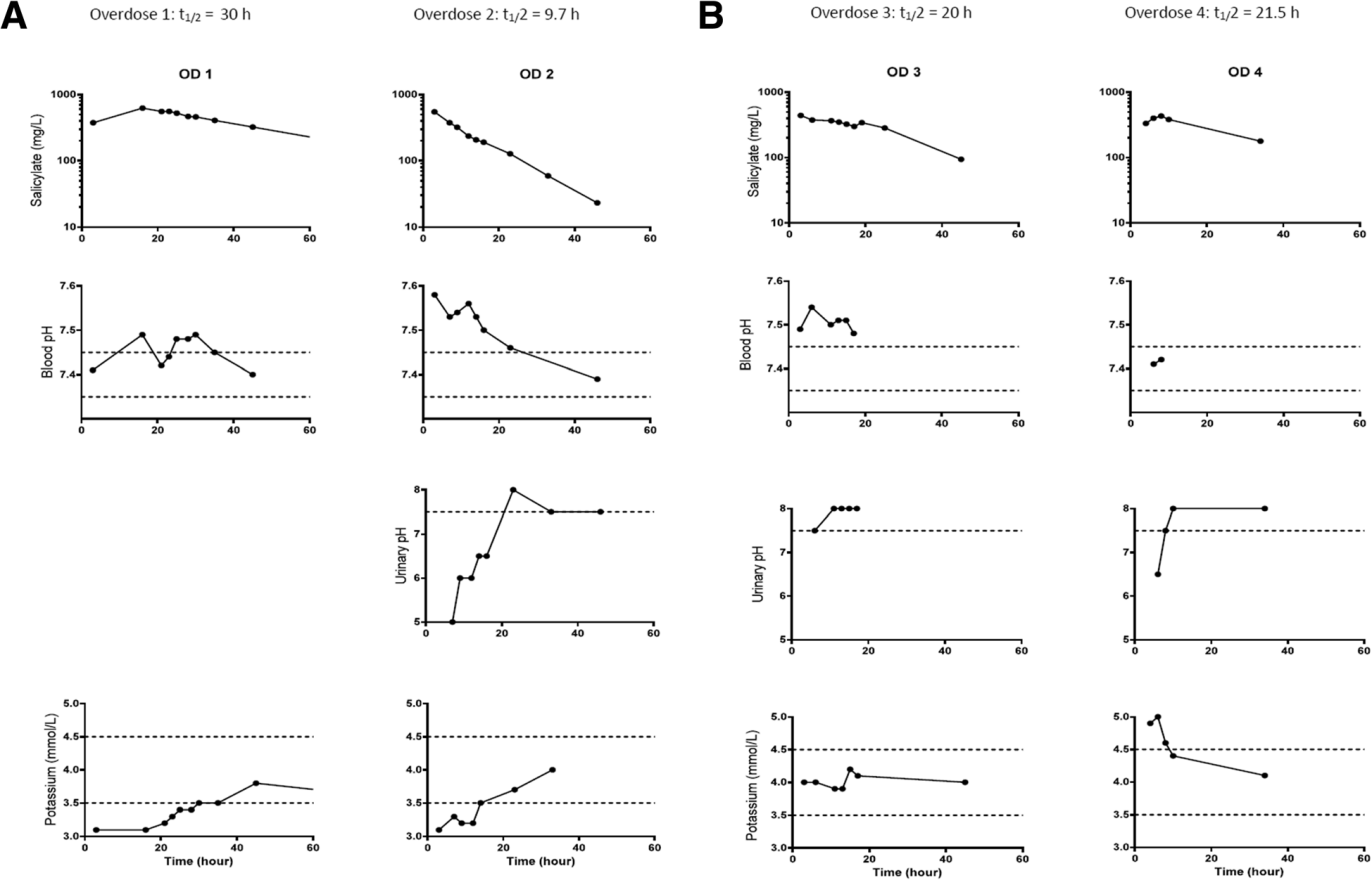
**Time courses of salicylate, pH (blood and urine) and plasma potassium following overdoses.** Graphed data for first and second overdoses (OD1 and OD2) **(A)** and third and fourth overdoses (OD3 and OD4) **(B)**, respectively. t_1/2_, Half-life. Areas between the dotted lines describe blood pH 7.35 to 7.45 and plasma potassium 3.5 to 4.5; Urinary pH dotted line represents 7.5.

Over the course of two months, a 75-year-old Caucasian man weighing 45kg who had a history of multiple previous drug overdoses, depression, bulimia and stage 3 chronic kidney disease [4] (creatinine between 160μmol/L and 140μmol/L, that is, estimated glomerular filtration rate, 39ml/min/1.73m^2^ to 46ml/min/1.73m^2^) presented on three occasions to St Vincent’s Hospital following aspirin overdoses. During his third admission, he overdosed with aspirin while on the ward recovering from the previous aspirin overdose. His alleged ingested dosages Table 1 were classed as “potentially lethal” in two episodes (>500mg/kg in both) and “serious” in the other two (300mg/kg to 500mg/kg) [2]. However, the peak plasma concentrations (Table 1) indicated a “moderate” severity of poisoning after the first and second overdose and “mild” severity after the other two [5]. The patient was dehydrated upon each admission with mild hyperventilation (respiratory rate around 30 breaths/min), nausea and tinnitus. He had no other neurological symptoms or signs. He was normothermic upon all admissions. In the second and third overdoses, the patient presented with respiratory alkalosis (Table 1, Figures 1A and 1B). Raised anion gap levels (that is, anion gap >18mmol/L) were observed upon the first admission (anion gap, 21mmol/L) and the second admission (anion gap, 22mmol/L) and resolved within 24 hours of treatment both times.In each of the four episodes, the patient received intravenous fluids (0.9% saline and/or 4% dextrose diluted 1:5 in saline) with the aim of achieving clinical euvolemia. Potassium supplementation was needed in the first, second and third overdoses. Additionally, sodium bicarbonate was administered after the second, third and fourth overdoses. The bicarbonate therapy led to an increase in urinary pH to between 7.5 and 8 (Figures 1A and 1B) (measured using Siemens Multistix 10 SG (Siemens Healthcare Diagnostics, Tarrytown, NY, USA) (referred to as dipstick below)). Single-dose charcoal was given after the second overdose only. None of the admissions warranted airway support, and there was no incidence of bleeding.

**Table 1:** Features of four overdoses of aspirin in an individual patient

**Overdose**	**Aspirin allegedly ingested, g (mg/kg)**	**Peak salicylate concentration (mg/L)**	**Admission plasma creatinine (μmol/L)**	**Creatinine 24 hours post admission (μmol/L)**	**Admission plasma potassium (mmol/L)**	**Admission blood pH**	**Potassium replaced (KCl each 24 hours) (mmol)**	**Input/output (each 24 hours) (L)**	**Bicarbonate (mmol/h)**
1	27 (600)	624	175	165	3.1	7.41	60	4.5/1.3	No
							40	3/2.7	
								2.5/2.8	
2	27 (600)	548	180	133	3.1	7.58	80	9.02/3.2	25 for 4h then 40 for 5h
								1.9/2.3	
3	22.4 (498)	441	141	159	4	7.49	20	3.38/2.97	30 for 10h
								2.5/1.1	
								1.12/no data	
4	14.4 (320)	432	142	142	4.9		–	5.3/1.1 + missing data	60 bolus then 25 for 3h

This patient had short high-dependency unit admissions (<48 hours) after the first, second and third overdoses. His fourth overdose was treated in a general ward. Refractory hypokalemia was encountered after the first and second overdoses, taking more than 20 hours to achieve normokalemia (Figure 1A and Table 1). Urinary pH between 7.5 and 8 was observed only when plasma potassium concentration was ≥3.7mmol/L (Figures 1A and 1B). Subsequently, using urine samples from one healthy volunteer (author DG) across a manipulated pH range of 5.0 to 8.5, dipstick readings were compared to those determined using a pH meter (Mettler Toledo, Columbus, OH, USA). The dipstick readings were consistently lower, by 0.36±0.2 units (based on six readings), than the “gold standard” pH meter readings.

The longest apparent half-life (t_1/2_) of elimination of salicylate (t_1/2_ = 30 hours) was seen after the first overdose, when urinary alkalization was not attempted (Figures 1A and 1B). The most rapid elimination was observed during the second admission (t_1/2_ = 9.7 hours). On that occasion, compared to the other overdoses, a much greater volume of intravenous fluid was administered, along with bicarbonate therapy and a single dose of charcoal (Table 1, Figures 1A and 1B).

The patient’s lengths of stay were 21 days (first overdose), 8 days (second overdose) and 6 days (third and fourth overdoses), respectively.

## Discussion

There is no antidote for aspirin overdose. The goals of therapy are to limit absorption of aspirin, to enhance elimination of salicylate and to provide supportive care [6]. Guidelines direct that emergency intervention may be required to achieve the following aims:

1. *Stabilize the airway and breathing*: Mechanical ventilation, if required, should be adjusted to maintain blood alkalosis (for example, hyperventilation to decrease partial pressure of carbon dioxide) [7].

2. *Treat dehydration, acid–base disturbance, hypokalemia, pulmonary edema, neuroglycopenia or seizure*.

Features of the toxicity of salicylate and the management of our patient were generally in accord with findings and recommendations for the management of aspirin overdose. However, variations in the time course of plasma salicylate concentrations may be related to the variable approaches used to treat individual episodes, as outlined below.

• *The stated dose of aspirin often does not indicate the potential level of toxicity*[1]. Our present case illustrates that the clinical features of severity correlated better with the plasma concentration of salicylate than the reported dosage of aspirin ingested.

• *Measurement of plasma concentrations of salicylate should be repeated over several hours because of the slow and erratic absorption of aspirin*[1]. Following all four of our patient’s overdoses, his plasma salicylate concentration was measured within one-half hour of admission and repeated at intervals during the management period. On the third occasion, a small, isolated rise in plasma concentration of salicylate was noted (299mg/L to 342mg/L) at 19 hours after ingestion of aspirin, with subsequent concentrations continuing to decline (Figure 1B).

• *Gastrointestinal decontamination with activated charcoal is recommended*[7]*when given early (after ingestion) and when the risk of aspiration is very low*. A single dose of charcoal was administered only after the second overdose, although the risk of aspiration was low on all occasions. Charcoal may have been useful in reducing the plasma concentrations of salicylate more rapidly in the other three episodes [8].

• *Hypovolemia should be corrected because intravascular volume depletion decreases salicylate excretion*[7]. The patient received intravenous fluids for each episode. He presented with the poorest renal function following the second overdose and was treated then with the largest amount of intravenous fluid. This was associated with the greatest fall in plasma creatinine within the first 24 hours of any of the four episodes (Table 1). Some of this decrease was most likely dilutional. Fluid balance is more critical in patients with baseline chronic kidney disease dictating the need for close hemodynamic monitoring.

• *Alkalization of urine by administering sodium bicarbonate is advised in order to increase the rate of excretion of salicylate*[9]. Titration of urinary pH to between 7.5 and 8 is commonly used to indicate successful alkalization. As mentioned above, urinary dipstick estimation of pH may underestimate the degree of alkalization. Bicarbonate administration in our patient was associated with a shorter half-life of salicylate in the second, third and fourth overdoses compared to the first overdose, when bicarbonate was not administered (Table 1, Figures 1A and 1B).

The half-life of salicylate at common analgesic doses of aspirin (300mg to 600mg every four to six hours) is two to four hours, but may increase to as much as 20 hours following overdose [7]. The half-lives of salicylate in our patient were therefore within the expected range for the second, third and fourth overdoses (t_1/2_=10 to 21 hours), but not in the first overdose (t_1/2_=30 hours), perhaps because bicarbonate was not used (Figures 1A and 1B).

• *Hypokalemia should be corrected to achieve effective urinary alkalization with bicarbonate*[7]*.* The importance of this point is illustrated in the second admission, when hypokalemia, despite potassium administration, was associated with failure to alkalinize urine for more than 20 hours (Figure 1A and Table 1).

• *Toxicity of salicylate may be greater in the elderly and infants than in young to middle-aged adults*[10]*.* Our patient was 75 years old, and thus an increased sensitivity to aspirin may have been expected.

• *Dialysis is indicated if metabolic acidosis is profound and is resistant to therapy and/or central nervous system toxicity is evident.* None of the overdoses in our patient appeared to be life-threatening on the basis of the clinical and laboratory features observed. The patient’s fluid balance (Table 1) indicated that there was no progression to oliguria or anuria.

There was heterogeneity in the approaches used to manage each episode of overdose in this patient. The patient had significant baseline renal impairment, which might have affected his responses to treatment. Urinary pH measured by using a dipstick can be misleading.

## Conclusion

Intravenous fluid hydration with the aim of achieving euvolemia was the intervention common to all four aspirin overdose episodes in our patient. Activated charcoal might have helped to decrease absorption [9]. Although urinary alkalization appeared to have increased the clearance of salicylate, it was the combination of treatments, best demonstrated in the management of the second overdose, which, we believe, optimized detoxification. These measures— oral charcoal, intravenous hydration, potassium replacement and urinary alkalization—were most effective in our patient, who had stage 3 chronic kidney disease.

Our experience with treating these four overdoses also reveals the importance of monitoring the implementation of management guidelines, as considerable unexplained variation in management was observed.

## Consent

Written informed consent was obtained from the patient for publication of this case report and any accompanying images. Also, approval to write up this case was obtained from the St Vincent’s Hospital Research and Ethics Committee. A copy of the written consent is available for review by the Editor-in-Chief of this journal.

## Competing interests

The authors declare that they have no competing interests.

## Authors’ contributions

DG, KW, GG, PN, HB and RD devised the study. DG performed the literature review, data collection and interpretation, review of the patient’s clinical course, manuscript drafting and project coordination. KW, GG, PN, HB and RD were involved in supervision and critical revision of the manuscript. All authors read and approved the final manuscript.
